# 

**Published:** 1895-02-16

**Authors:** 


**Notices of Births, Deaths, and Marriages** are inserted in this column at a charge of 2s. 6d. for an announcement not exceeding four lines.

**Notice to Advertisers and Subscribers.**

All Advertisements, Orders for Copies of The Hospital and Business Communications should be addressed to **The Manager, NOT TO THE EDITOR,** The Hospital, 428, Strand, London, W.C.

The Cost of Subscription, post free, is as follows:—Per week, 2½d.; per quarter, 2s. 8d.; per half-year, 5s. 3d.; per annum, 10s. 6d. Foreign:—Per Annum, 15s. 2d.; payable, in all cases, in advance.

### NOTICE TO CORRESPONDENTS.

All MS., Letters, Books for Review, and other matters intended for the Editor should be addressed The Editor, The Lodge, Porchester Square, London, W.

The Editor cannot undertake to return rejected MS., even when accompanied by stamped directed envelope.

**"Burdett's Hospital Annual," 1895,**

In Active Preparation.

Sixth year of publication. About 900 pp. Scarlet cloth, gilt lettered Price 5s. A most complete guide to British, Colonial, Indian, and American Hospitals, Dispensaries, Nursing and Convalescent Institutions, and Asylums. A few copies of the "Annual" for 1894 may still be obtained on application to the Publishers, The Scientific Press (Limited), 428, Strand, London, W.C.

**NOTICE.—The Index for Vol. XVI. (ending September 24th, 1894) is on sale at the Office of this Journal, Price 2½d., post free.**

### Scraps and Gleanings.

The accounts of the Chelmsford Infirmary for the past year show a falling off of about £100.

A gift of 15 brace of pheasants has been received at Guy's Hospital from Her Majesty the Queen.

We are glad to see the subscription list of the Worcester Ophthalmic Hospital is being well maintained.

A ball in aid of the building fund of the Royal Free Hospital will be held at the Westminster Town Hall on the 20th inst.

Sir Ralph Thompson has been elected chairman of the board of the Middlesex Hospital, in the room of Lord Sandhurst.

The Cottage Hospital of Ottery St. Mary's shows an excellent balance-sheet in this, the first year of inauguration of the institution.

The Hospital for Infectious Diseases at Bangor has now been officially opened, Lord and Lady Penrhyn being present at the ceremony.

Since the Working Men's Committee has been formed for the Belfast Royal Hospital the annual income from this source has increased by over £800.

The Duke of Cambridge will preside over the festival dinner of the Hospital for Children, Great Ormond Street, at the Hotel Metropole on May 17th.

The annual meeting of subscribers of the Eastern Dispensary, Bath took place recently. The report is a good one, and the accounts show, a favourable balance in hand.

It is proposed to increase the Homœopathic Hospital at Tunbridge Wells, so soon as funds make this feasible. A legacy of £50 has been received by the committee of this institution.

The total expenditure of the Ashburton and Buckfastleigh Cottage Hospital shows an expenditure this year of £202 on the working expenses; this sum shows a saving under every head.

The heating apparatus of the New County Combination Hospital, Alva, has to be entirely overhauled through the recent bursting of the hot-water pipes. The damage done necessitates an outlay of £90.

Many improvements have been effected at the Rotherham Hospital and Dispensary during the past year. The drainage system has been overhauled, and hot-water pipes have been laid down, necessitating a new boiler.

The Harrogate Cottage Hospital authorities are shortly proceeding with the work of enlarging the hospital at an estimated cost of about £2,000, and will appropriate the £322, the Clarence Ward Fund, collected by Mrs. James Myrtle.

We learn that the Committee of the Hahneman Homœopathic Hospital at Liverpool record their satisfaction at the fact that several cases of chronic alcoholism have been admitted to the wards with more than a temporary benefit being derived.

A first instalment of £250 from the Cotton District Convalescent Fund has been paid on account of the building in course of erection at the Southport Convalescent Hospital; the accommodation for nurses and servants is not completed yet.

The Duke of Connaught and the Duke of Cambridge have each subscribed three guineas to the Women's Convalescent Home Association, that free letters for the homes may be placed at the disposal of the Matron of the Army Clothing Depot in Pimlico.

Lady Kortright, of Roden House, Brentwood, has sent the munificent gift of £7,000 to the treasurer of the Grosvenor Hospital for Women and Children. This is in addition to a sum of £1,000 already contributed by Lady Kortright. Both sums are in aid of the building fund.

At the annual meeting of the Helston Public Dispensary it was suggested that if Mr. Passmore Edwards munificent offer of providing a public library at Helston should fall through, he should be approached with a view of building a cottage hospital, which is much needed in the locality.

A Paris correspondent telegraphs: A disease little, if at all, known in England is very prevalent here. This is purulent pneumonia; and the illness originated in a cargo of green parrots landed some years ago at Bordeaux. The birds brought the malady to the different towns they were sent to.

The Kynoch wards to the Turner Memorial, Keith, are now completed and furnished, through the generosity of Mr. and Mrs. Bryson, of Strathlene. Besides this, the same benefactors have provided handsome enclosing walls round the garden of the institution, a new approach, and new walks.

The debit balance in the accounts of the Birkenhead Borough Hospital has increased by £228 this year, although the strictest economy has been exercised, with the result that the expenditure was £200 less than the previous year. No further reductions can possibly be made without closing some of the wards.

A generous offer has been made to the authorities of the Surrey County Hospital, Guildford. Mr. T. Rees offers to supply the hospital with all the coal it consumes, at the absolute bare net cost to him in Guildford Yard. With this he would wish to send an invoice of the cost of the coal at the pit, charges for tolls, &c.

During the year 4,225 cases have been treated at the Royal Victoria Hospital, Bournemouth. It was pointed out at the last annual meeting that £1,000 ought not to represent the capacity of the town for subscriptions. The committee having now thrown open their advantages to the district around, are claiming also a larger area for contributions.

The work of the Brighton Lying-in Institution continues to increase. The number of hospital pupils has reached the full extent of accommodation provided for them. Six of these students passed the examination of the London Obstetrical Society last autumn. The result of the re-arrangement of the nursing and domestic staff has been all that was desired.

At the 78th annual meeting of the Royal Ear Hospital, the medical board reported that a great increase in the number of patients rendered it necessary to enlarge the present accommodation, and the committee recommended that new premises be built at a cost of £20,000, towards which several friends of the institution have promised to contribute.

### THE STUDENT OF MEDICINE.

### APPOINTMENTS.

S. R. Alexander, M.D.Lond., M.R.C.S., appointed Medical Officer and Public Vaccinator for the No. 1 District of the Faversham Union. A. E. Broster, L.R.C.P.Edin., M.R.C.S.Eng., reappointed Medical Officer for the Brassington District of the Ashbourne Union. L. A. Buck, M.R.C.S.Eng., L.S.A., appointed Resident Medical Officer for the Workhouse of the Bolton Union. H. Burland, F.R.C.S., L.R.C.P., appointed Medical Officer to the Finedon Urban Council. A. Donald, M.A., M.D., C.M.Edin., M.R.C.P.Lond., appointed Obstetric Physician to the Manchester Royal Infirmary. A. Hall, M.R.C.S.Eng., reappointed Medical Officer for the Calton District of the Ashbourne Union. R. W. Henry, M.B., B.Ch.Dubl.Univ., appointed Senior House Surgeon to the Birmingham and Midland Eye Hospital. J. E. Hickman, L.R.C.P.Lond., M.R.C.S.Eng., appointed Resident Medical Officer to the Guest Hospital, Dudley. E. R. Hutton, L.R.C.P.Lond., M.R.C.S.Eng., appointed Medical Officer for the 2nd Tottenham District of the Edmonton Union. A. E. Kempthorne, M.R.C.S., L.S.A., appointed Medical Officer for the No. 5 District of the Bethnal Green Union. P. R. Littleton, M.R.C.S.Eng., reappointed Medical Officer for the Ashbourne District of the Ashbourne Union. D. U. McLennan, M.D.Edin., appointed Medical Officer of Health for the Widnes Urban Sanitary District. G. B. Masson, L.R.C.P., L.R.C.S. Edin., appointed Medical Officer for the Fineham District of the Downham Union. A. D. Owen, M.R.C.S., L.R.C.P.Lond., L.S.A., appointed Divisional Surgeon, Metropolitan Police. R. Powell, L.R.C.P.Lond., L.S.A., appointed Medical Officer and Public Vaccinator for the Merthyr Cynog District of the Brecon Union. W. M. Robertshaw, M.B., C.M.Edin., reappointed Medical Officer of Health to the Stocksbridge Urban District Council. Mr. J. G. Robertson, appointed Medical Officer for the 3rd District of the Royston Union. C. F. Seville, M.B.Lond., appointed Medical Officer to the Helston Dispensary. R. H. Steen, B.A.R.U.I., M.B.Lond., appointed House Physician to the Brompton Hospital for Consumption and Diseases of the Chest. R. J. Swan, M.R. C.S.Eng., L.S.A.Lond., appointed Consulting Surgeon to the London Tramways Company. Mr. W. B. Tait, appointed Medical Officer for the 5th District of the Ashton-under-Lyne Union. W. Wearne, M.R.C.S.Eng., L.S.A., appointed Medical Officer to the Helston Dispensary. E. S. Wood, L.R.C.P., L.M., M.R.C.S.Eng., appointed Factory Surgeon for Cambridge and District.

### VACANCIES.

The following vacancies are announced this week, the amount of salary in each case being given after the name of the institution:

### Resident Medical Officer.

General Infirmary, Leeds.—Resident Casualty Officer. Salary £100 per annum, with board, lodging, and washing. Applications to Mr. Littlewood, Secretary of the Faculty, General Infirmary, Leeds, by February 20th.

### Non-Resident Medical Officers.

Derbyshire Royal Infirmary, Derby.—Honorary Physician, Honorary Surgeon, Honorary Consulting Dental Surgeon. Applications to Walter G. Carnt, Secretary, by February 18th.

Kent and Canterbury Hospital, Canterbury.—Surgeon. Applications to the Secretary by February 19th.

Taunton and Somerset Hospital.—Honorary Surgeon. Must reside within two miles of the hospital. Applications to J. H. Biddulph Pinchard, Secretary, 13, Hammet Street, Taunton, by February 16th.

### THE STUDENT OF MEDICINE—*continued.*

Westminster Hospital, Broad Sanctuary, S.W.—Fourth Assistant Physician, must be Fellow or Member of the Royal College of Physicians of London, not practising pharmacy and midwifery. Applications to the Secretary before February 26th.

### TRIPLE QUALIFICATION.

Second Examination.

ADVANCED ANATOMY.

(All the questions to be answered.)

1. Mention, in their order, the various parts that are in contact with the lower jaw-bone.
2. (*a*) Mention where valves are met with in the heart and in the blood-vessels of the thorax; and (*b*) describe the anatomy and adaptation of those found at the apertures of the left ventricle.
3. Describe the ischio-rectal fossa—its boundaries and contents, giving the appearances presented by these parts in dissection.
4. (*a*) Mention the muscles by which each of the movements of the shoulder joint is effected; and (*b*) give the attachments of the muscles that lie in contact with the capsule of that joint.

PHYSIOLOGY.

(All to be answered.)

1. Describe the minute structure of a tooth. State the nature, number, and usual date of appearance of the teeth of the first dentition.
2. Describe the varieties of endings of sensory nerves, mentioning any situations in which the various endings are found.
3. State the nature of the exchanges between the blood in the pulmonary capillaries and the air in the pulmonary alveoli.
4. What are the channels of absorption in the gastric mucous membrane, and the mucous membrane of the small intestine? What is the nature of the absorbed materials in each case, and what is their subsequent course?

MATERIA MEDICA.

(All to be answered.)

1. What is the composition and what the dose of: Acidum sulphuricum aromaticum, decoctum aloes compositum, pilula hydrargyri subchloridi composita, pulvis glycyrrhizæ compositus?
2. Give a short account of the compounding of pills, referring, *inter alia*, to the special character of the excipient as required by the particular drug.
3. Describe three of the tap-shaped roots of the pharmacopœia. Name the preparations of each root and their doses.
4. Name the preparations of conii folia and state the mode of preparing the extract.

ELEMENTARY ANATOMY.

(All the questions to be answered.)

1. Enumerate the bones articulating with the temporal bone.
2. Give the origins, insertions, and nerve supply of the pronators and supinators of the forearm.
3. Describe the ligaments of the ankle joint.
4. State the relative positions of the structures exposed on reflecting the gluteus maximus muscle.

